# Structural and functional analysis of lysozyme after treatment with dielectric barrier discharge plasma and atmospheric pressure plasma jet

**DOI:** 10.1038/s41598-017-01030-w

**Published:** 2017-04-21

**Authors:** Sooho Choi, Pankaj Attri, Inhwan Lee, Jeongmin Oh, Ji-Hye Yun, Ji Hoon Park, Eun Ha Choi, Weontae Lee

**Affiliations:** 1 Department of Biochemistry, College of Life Science & Biotechnology, Yonsei University, Seoul, 120-749 Korea; 2 Plasma Bioscience Research Center/Department of Electrical and Biological Physics, Kwangwoon University, Seoul, 139-701 Korea

## Abstract

The variation in the biological function of proteins plays an important role in plasma medicine and sterilization. Several non-thermal plasma sources with different feeding gases are used worldwide for plasma treatment, including dielectric barrier discharge (DBD) and atmospheric-pressure plasma jet (APPJ) as the most commonly used sources. Therefore, in the present work, we used both DBD and APPJ plasma sources with N_2_ and air as feeding gases to evaluate the effects on the structural, thermodynamic, and activity changes of enzymes. In the current work, we used lysozyme as a model enzyme and verified the structural changes using circular dichroism (CD), fluorescence, and X-ray crystallography. In addition, we investigated the lysozyme thermodynamics using CD thermal analysis and changes in the B-factor from X-ray crystallography. The results showed that lysozyme activity decreased after the plasma treatment. From these analyses, we concluded that N_2_-feeding gas plasma disturbs the structure and activity of lysozyme more than Air feeding gas plasma in our experimental studies. This study provides novel fundamental information on the changes to enzymes upon plasma treatment, which has been absent from the literature until now.

## Introduction

The production of highly reactive species (RS) such as reactive nitrogen species (RNS) and reactive oxygen species (ROS) by plasma at low temperature provides various advantages for flexible operations in various fields^1–4^. During atmospheric-pressure plasma/cold plasma exposure, the RNS and ROS, collectively termed RONS, can move from the gas to solution phase^5, 6^. These gas-phase RONS can activate or generate new RONS in the liquid by various reactions^7, 8^. In particular, RONS generated by plasma in solution, either indirectly or directly, have attracted substantial attention in various fields such as for plasma sterilization, blood coagulation, living-tissue treatment, induction of apoptosis, cancer therapy, and cell proliferation^9–23^. When generated in solution by plasma, these RS can modify or activate the biological constituents (i.e. cell membrane, lipids, proteins, and other molecules) and cell media. Modification or oxidation of the various components interacting with cell membrane receptors results in the transfer of diverse signaling processes that can affect a wide range of cellular activities, including cell differentiation, cell proliferation, cell migration, deactivation of bacteria, anticancer activity, and wound healing^6^. To understand these applications of plasma, numerous probable mechanisms have been suggested^19–21^, however, no proper hypothesis has been put forward, and the precise mechanism is still elusive. Biological functions are mainly affected by proteins/enzymes, as one of the main components of the cells and tissues. A few studies have been conducted to determine the effects of plasma on proteins by our group and others^24–31^.

Therefore, to understand the mechanism of cold plasma on the structural changes and enzymatic activity, we have used lysozyme as a model protein. Lysozyme is extensively used in pharmaceuticals industries due to its anti-inflammatory, immune modulatory and anti-tumor properties^32–34^. Lysozyme is formed by 129 tactic amino acid residues that contain four disulfide bonds, three tyrosine (Tyr) and six tryptophan (Trp) group. Trp62 and Trp108 are organized near the substrate binding site that plays important role in lysozyme binding with a substrate/inhibitor and help in stabilizing the native structure^35^. This offers the chance of associating lysozyme dynamics with enzymatic activity that could provide information on the ligand-induced conformational change and lysozyme–ligand interaction around the binding site^36^.

To gain insight about the mechanism it is necessary to study the action of plasma on molecular level. Moreover, until now no study has shown that what type of exact structural changes in proteins/enzyme occurred after the plasma treatment, what is the role of using different gases and different devices. Therefore, in this work we have used two types of plasma sources, dielectric barrier discharge (DBD) plasma and atmospheric pressure plasma jet (APPJ) with N_2_ and Air as feeding gases, but without changing the distance of treatment and plasma characteristics [gas flow rate, treatment time and voltage]. For this study, we used Lysozyme as a model enzyme and checked the structural changes using circular dichroism (CD), fluorescence and X-ray crystallography. Additionally, we have checked the thermodynamics of protein using the thermal analysis by CD and analysis of B-factor by X-ray crystallography. Later, we studied the lysozyme activity after the DBD and APPJ treatments using different feeding gases.

## Results

Numerous studies emphasize the effect of RONS created by cold plasma sources on cells and tissues. Moreover, a few recent studies have evaluated the effect of plasma on proteins/enzymes. However, the specific mechanism has not been proposed yet in the literature. Therefore, in the present study, we treated lysozyme with DBD and APPJ using N_2_ and air as the feeding gases for 8 min and 12 min, respectively, in phosphate buffer.

### Analysis of reactive species generated in solution and in the gas phase using a chemical method and optical emission spectroscopy

**Figure 1:**
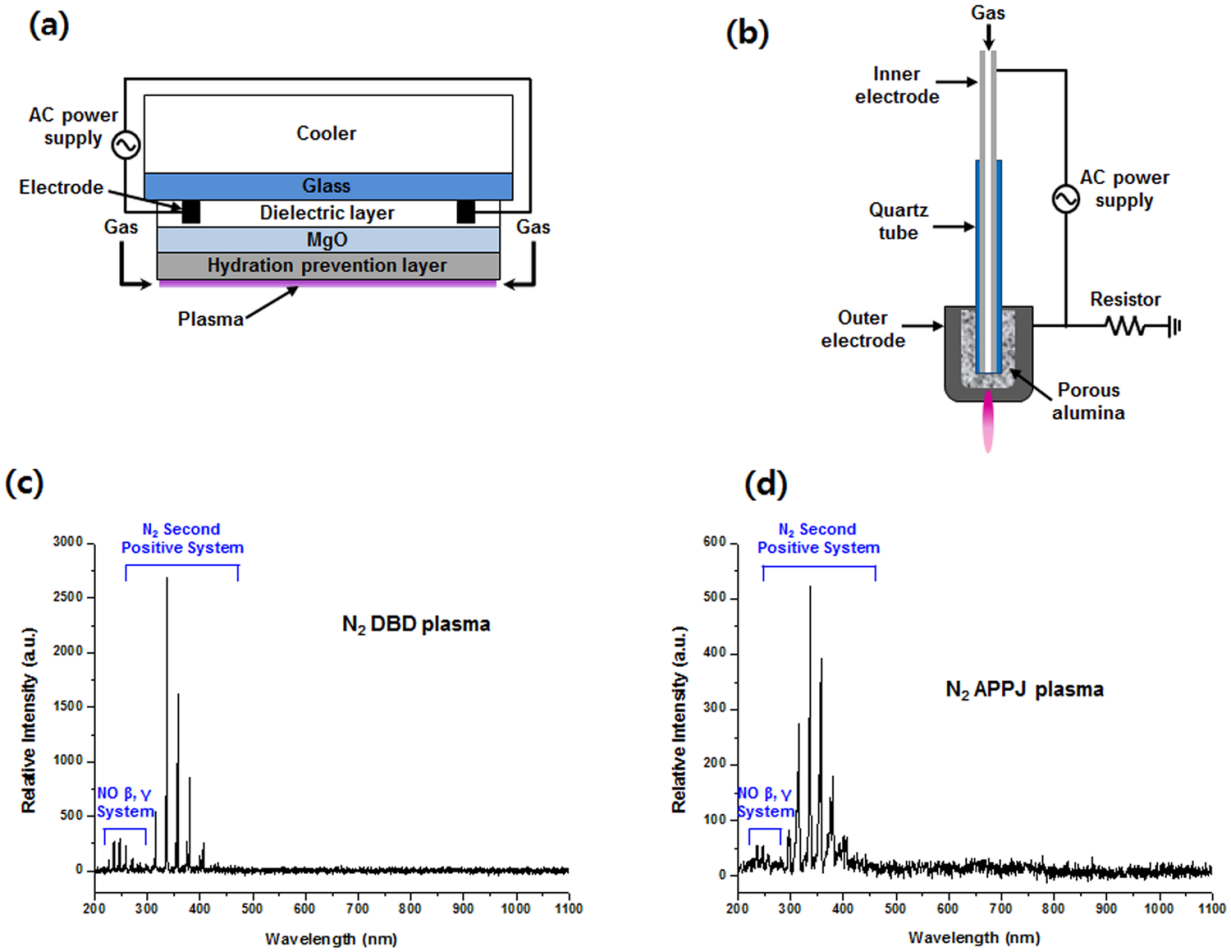
Schematic depiction and optical emission spectra. Schematic depiction of (**a**) dielectric barrier discharge (DBD) and (**b**) atmospheric pressure plasma jet (APPJ) are shown. Optical emission spectra of (**c**) the N_2_-DBD plasma and (**d**) the N_2_-APPJ were observed, respectively.

The Fig. 1a,b, shows the schematic representation of the DBD and APPJ at atmospheric pressure. We analyzed some important RS generated by the DBD and APPJ plasma in the presence of feeding gases (N_2_ and Air) for 12 min, without changing the treatment distance (distance between the plasma sources edge and treatment solution) and plasma characteristics such as gas flow rate, treatment time and voltage, as shown in Figure S1. The energy of DBD with N_2_ and Air feeding gases were 0.5 and 0.8 J/s, respectively. Whereas, the energy of APPJ with N_2_ and Air feeding gases were 0.3 and 0.4 J/s, respectively. In the buffer solution, we measured the production of OH, H_2_O_2_, and NO radicals. We detected that the highest level of OH and H_2_O_2_ radicals in N_2_ plasma and lowest in Air plasma, for both plasma sources. To compare the generation of OH radicals using different feeding gases with different plasma devices (DBD and APPJ), we have performed the TA (terephthalic acid) test as shown in Figure S1a. Figure S1a, shows that the fluorescence intensity of hydroxy terephthalate ions formed at ≈425 nm was higher with N_2_-APPJ treatment than with Air-APPJ, this reveals that more OH radicals were generated in N_2_-APPJ than in Air-APPJ plasma. Similar results were observed for the DBD with N_2_ and Air as the feeding gases. Hence, N_2_ as the feeding gas for both plasma sources can produce more OH radicals than Air as a feeding gas, as seen in Figure S1a, although the discharge energy was highest for the Air-plasma than for N_2_-plasma. But the NO concentration was highest in Air as feeding gas as compared with the N_2_ as the feeding gas for both plasma devices. Additionally, we have studied the changes in pH and temperature of the buffer solution after 12 min treatment of DBD and APPJ for both feeding gases, as shown in Figure S2.

The pH was decreased for the Air as the feeding gas and slightly increased for N_2_ as the feeding gas for both sources, but the change was not significant enough to cause a structural distortion of lysozymes. Besides, we have recorded the optical emission spectra (OES) for DBD and APPJ treatment for both feeding gases (N_2_ and Air) to know the types of short lived radicals formed during the treatment. Strong emission lines were observed for the molecular NO β, γ system for N_2_ feeding gas for both DBD and APPJ as shown in Fig. 1c,d. Whereas weak lines between 200 and 250 nm were observed for the Air plasma for both DBD and APPJ plasma, as shown in Figure S3. The number of electrons decreased for Air plasma because O_2_ is a highly electronegative gas present in Air and it decrease the number of electrons by attachment, which resulted formation of O^−^ and O_2_^−^ ions. The strong peak of atomic oxygen at ≈777.5 nm was observed for Air APPJ, but was weak for the DBD plasma, possible due to the difference in energy of the two plasma sources.

### CD and Fluorescence analysis

**Figure 2:**
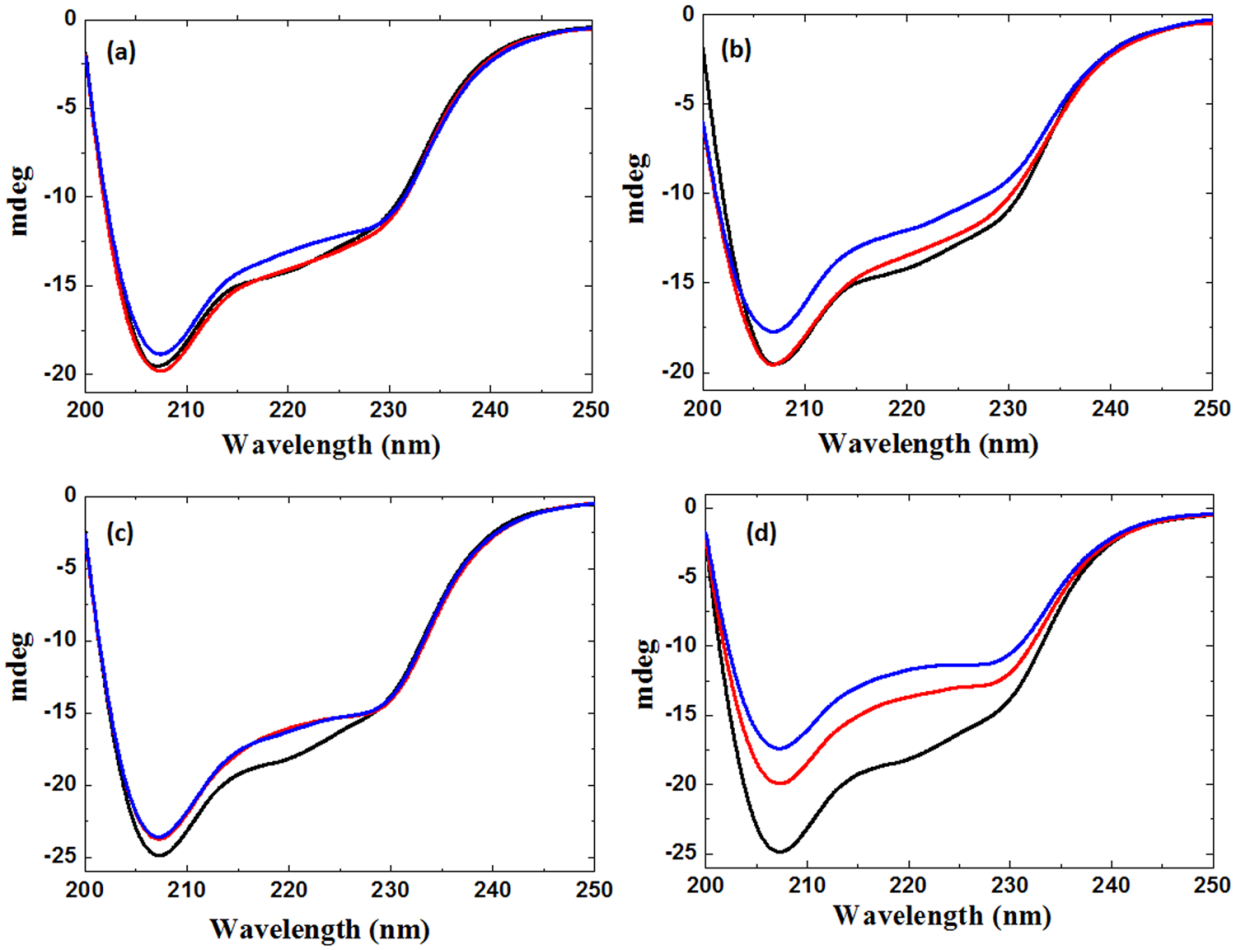
CD spectrum after plasma treatment. The far-UV CD spectrum of lysozyme were acquired for (**a**) Air-DBD; (**b**) N_2_-DBD; (**c**) Air-APPJ and (**d**) N_2_-APPJ treatment for 8 min (red) and 12 min (blue).

To gain a more detailed understanding of the structural modifications to lysozyme using DBD and APPJ plasma for 8 min and 12 min, we performed CD experiments^29^. The far-UV CD spectra of lysozyme indicated a distortion of the secondary structure in the different plasma sources as well the different feeding gases. The spectra in Fig. 2, shows a negative peak at ≈208 nm resulting from a change in the proportion of the α-helix and a shoulder at ≈18 nm indicating a change in the proportion of the β -sheet structure in the enzyme. The results in Table S1 clearly reveal that during the DBD treatment the α-helix structure decreases and β-sheet increases for the lysozyme. Figure 2 and Table S1, shows that for DBD treatment with Air as feeding gas for the 8 min and 12 min, the α-helix decreases to 49.7% and 48.3% respectively. Whereas, for N_2_ gas DBD treatment for 8 min and 12 min, the α-helix decreases to 31.0% and 27.1% respectively. This shows that α-helical decreases as compared to the control lysozyme α-helix that is 51.3%. However, after the APPJ treatment for the 8 and 12 min, for both Air and N_2_ plasma, showed that the α-helix increases and β-sheet structure decreases. The higher decrease in β-sheet is observed for the N_2_-APPJ as compared with the Air-APPJ. The α-helix structure increased to 58.2% and 62.0%, in the presence of Air APPJ for 8 min and 12 min treatment, respectively. Whereas, for N_2_ gas APPJ treatment for 8 min and 12 min, the α-helical increases to 59.5% and 70.4% respectively. These results are quite different for both devices in which for the DBD the percentage of α-helix decreases, while for the APPJ the percentage of α-helix increases for the same enzyme.

**Figure 3:**
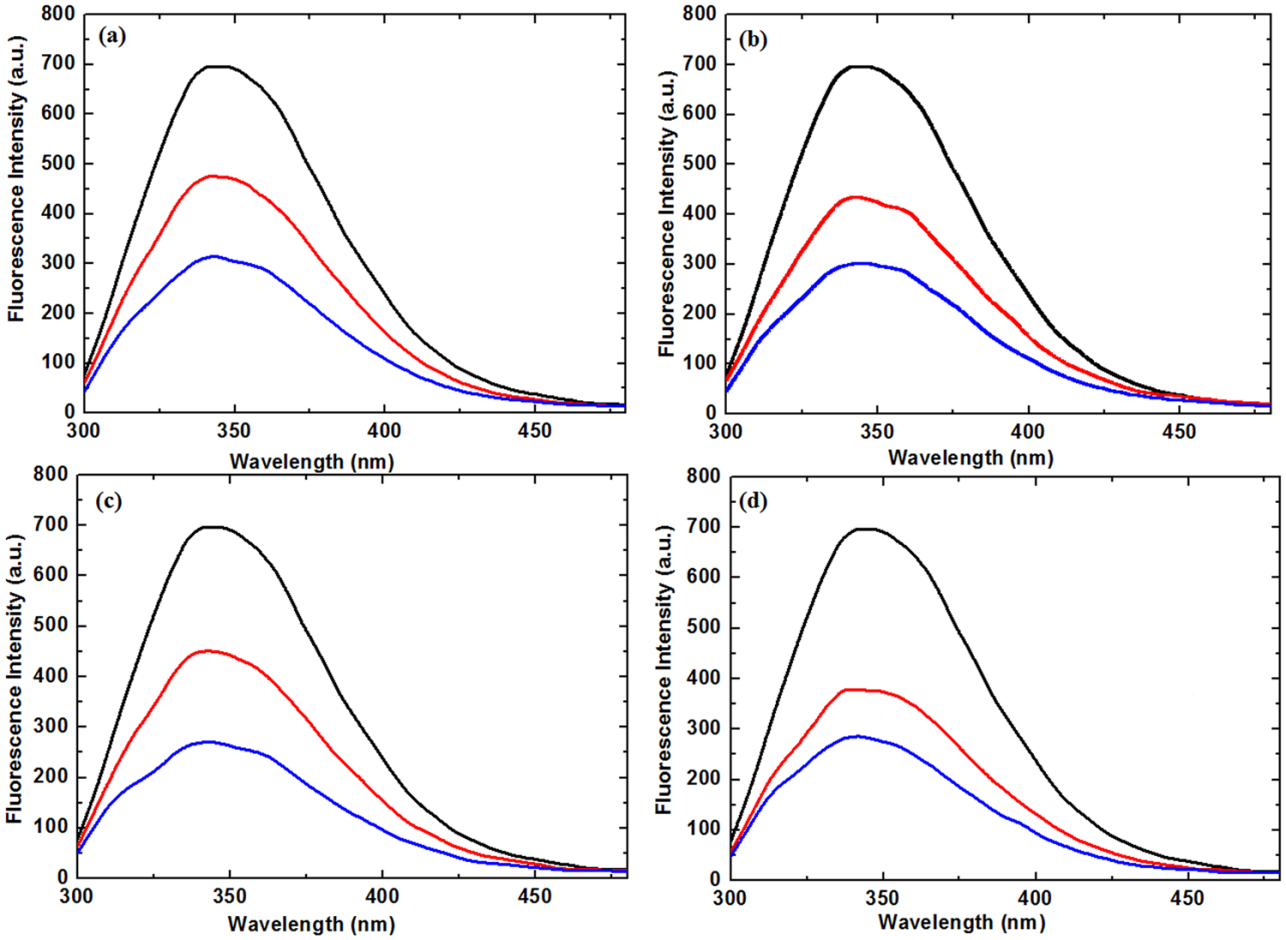
Fluorescence spectrum after plasma treatment. Spectrum were detected after (**a**) Air-DBD; (**b**) N_2_-DBD; (**c**) Air-APPJ and (**d**) N_2_-APPJ treatment for 8 min (red) and 12 min (blue).

Further, to investigate the structural changes of lysozyme induced by the treatments, we conducted fluorescence analysis^26, 29^, as shown in Fig. 3. The intrinsic tryptophan (Trp) emission intensity provides the change in the structural information of the aromatic environment of the lysozyme before and after plasma treatment. The fluorescence intensities depend on Trp environment as well as the structure of Trp. Therefore, the change in intensity or shifts in wavelength are due to the change in the solvent environment around the Trp residue in the enzymes. Mostly, the change in the polarity around the Trp is considered as decrease in the fluorescence intensities. The intrinsic Trp fluorescence of lysozyme after the plasma treatment is shown in the Fig. 3. Lysozyme showed intrinsic fluorescence from the Trp residues with excitation at 295 nm, with a decrease of fluorescence intensity at 340 nm after plasma treatment. However, we did not observe any shift in the maximum wavelength of the fluorescence spectra following plasma treatment with both feeding gases, irrespective of the device used.

### Structural analysis of the lysozyme by X-ray crystallography

**Figure 4:**
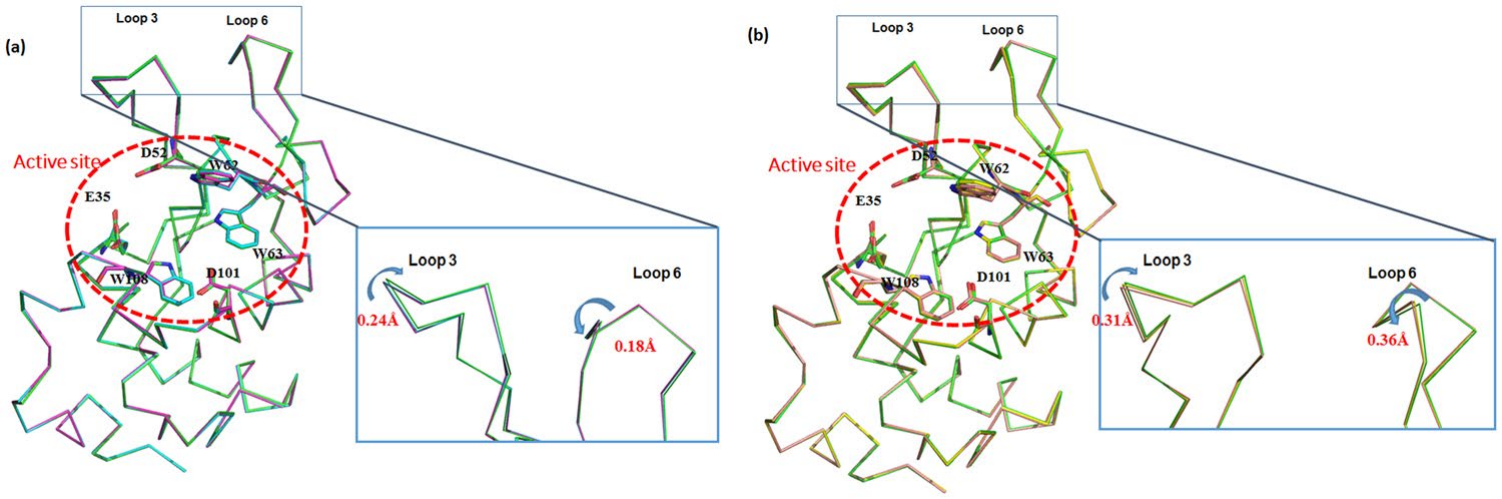
Backbone conformational changes of lysozyme. (**a**) The structural differences between control (magenta) and treatment (cyan: 8 min and green: 12 min) of Air-DBD. (**b**) The structural differences between control (salmon) and treatment (yellow: 8 min and green: 12 min) of N_2_-APPJ.

**Figure 5:**
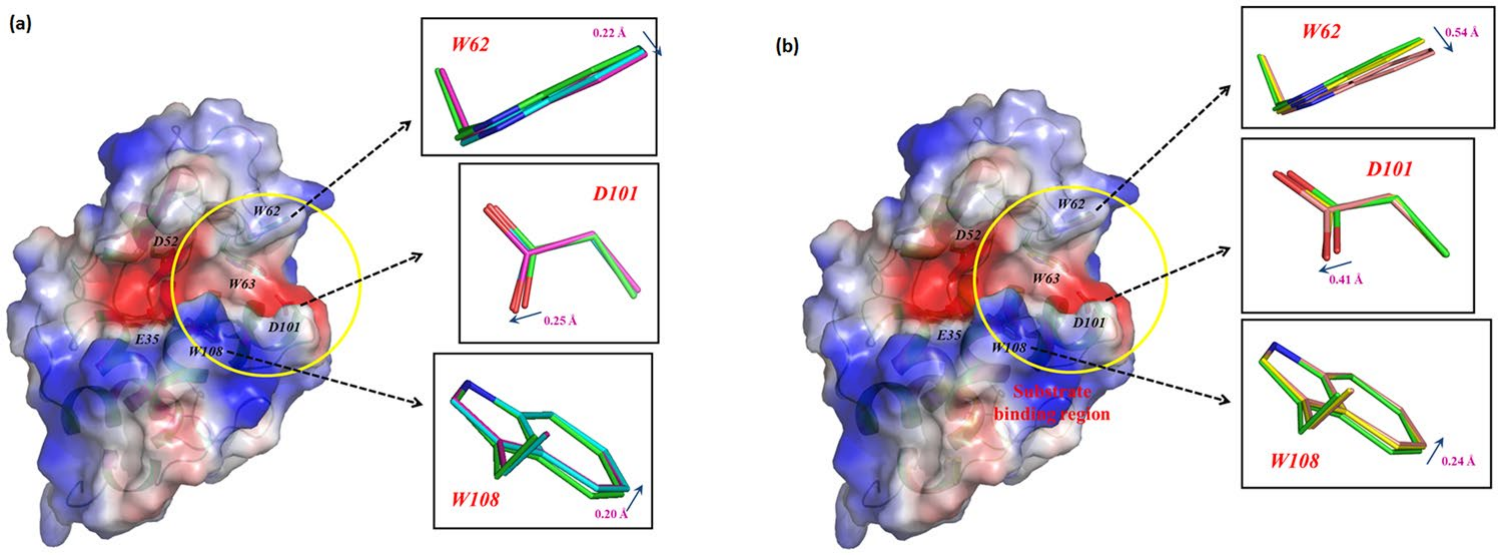
X-ray crystallographic structural change of substrate binding site. The structural differences between control and treatment (8 and 12 min) of (**a**) Air-DBD and (**b**) N_2_-APPJ are shown.

Three-dimensional structure analysis using X-ray crystallography can provide more detailed information about lysozyme to help understand the structure and activity of the enzyme^37^. The results described above clearly indicated that plasma treatment induced structural changes of lysozyme; however, the extent of the structural changes occurring during the refolding process remain unknown. Therefore, by looking at the structure of lysozyme by X-ray crystallography, we can better understand the plasma induced conformational deformation. For this study, we analyzed the lysozyme structure after treatment with Air-DBD (minimum structural changes observed with CD and fluorescence analysis) and N_2_-APPJ (maximum structural changes observed with CD and fluorescence analysis), as shown in Fig. 4. The structure of lysozyme after 8 min treatment with Air-DBD and N_2_-APPJ were overlaid with control (without plasma treatment), as shown in Fig. 4a and b. This may be possible due to refolding of lysozyme structure during the crystal formation. Whereas, the treatment of lysozyme with Air-DBD plasma for 12 min, we observed the structural changes at loop 3 and loop 6. Loop 3 is laying on β-turn 1 and loop 4 is laying in-between β2 and α3 (Fig. 4a). The shift of the loop 3 is about 0.24 Å after 12 min treatment as compared to the control. Similarly, lysozyme structure changes for the N_2_-APPJ treatment for 12 min, the affected part is the same as that of the Air-DBD (loop 3 and loop 6). Whereas, the distortion angle is more for the N_2_-APPJ that is about the 0.31 Å for loop 3 and 0.36 Å for the loop 6 (Fig. 4b). This showed that the affected amino acid was the same for the Air-DBD and N_2_-APPJ but the extent of distortion was different. This also supported our above data of CD and fluorescence that N_2_-APPJ had stronger action on the distortion of the lysozyme structure. Later, we checked the action of plasma on the active site or the substrate binding site. Here we also observed that for the 8 min treatment of Air-DBD and N_2_-APPJ there was no change in the structure of the substrate binding site, as compared to control (without plasma treatment). However, for 12 min treatment of the Air-DBD, we observed that W62 substrate binding site was changed to 0.22 Å, D101 substrate binding site was changed to 0.25 Å and W108 substrate binding site was changed to 0.20 Å, as shown in Fig. 5a. Moreover, when we checked the substrate binding site for N_2_-APPJ treatment for 12 min, we observed that W62, D101 and W108 substrate binding site was changed to 0.54 Å, 0.41 Å and 0.24 Å, respectively, as shown in Fig. 5b.

### Enzymatic activity of lysozyme before and after plasma treatment

To evaluate whether the structural changes which occurred in lysozyme can affect the enzymatic activity, we performed an activity test using *Micrococcus lysodeikticus cells* after the plasma treatment for 12 min in the presence of both feeding gases, as shown in Figure S4. The lysozyme assay is based on the concept that the degradation of the bacterial cell wall of *Micrococcus lysodeikticus* by lysozyme results in a decrease of the absorbance of the sample at 450 nm over time due to the change in turbidity of the suspension. The residual activity decreased with time, more activity was lost after the APPJ treatment as compared to the DBD treatment. And among all the feeding gases the N_2_-APPJ plasma had stronger action on the lysozyme activity followed by Air-APPJ, N_2_-DBD and Air-DBD (Figure S4). This activity data indicated that the reduction in the residual activity was due to physical/chemical modification or denaturation of the enzymes after the plasma treatment.

### Thermodynamic analysis and average B factor

The thermal stability of lysozyme is an important parameter for enzyme function; therefore, we conducted CD thermal analysis for each treatment. The thermodynamics of the lysozyme unfolding was evaluated at 222 nm, and the results are shown in Table S2. Chaffotte *et al*. reported that this wave length is close to the isodichroic point of native lysozyme and the refolding intermediate (formed after 4 ms of refolding)^38^. The results revealed a decrease in T_m_ values (the temperature at which 50% of the proteins are unfolded) of lysozyme for all feeding gases with both plasma sources (DBD and APPJ). The observed T_m_ of the lysozyme control without plasma treatment was found to be ≈78.12 °C based on CD thermal analysis test, whereas after the Air-DBD plasma treatment for 8 min and 12 min, the T_m_ decreased to ≈77.30 °C and 76.82 °C, respectively. After the Air-APPJ treatment for 8 and 12 min, the T_m_ decreased to ≈77.25 °C and 75.85 °C, respectively. Moreover, the change in T_m_ after for N_2_-DBD treatment for the 8 and 12 min was ≈76.12 °C and 74.91 °C, respectively. In addition, the T_m_ decreased for lysozyme after the N_2_-APPJ treatment for the 8 and 12 min to ≈76.09 °C and 74.01 °C, respectively. Hence, these results demonstrated that N_2_-APPJ or N_2_-DBD has a greater effect on the thermodynamics as compared to the Air-APPJ or Air-DBD treatments.

**Figure 6:**
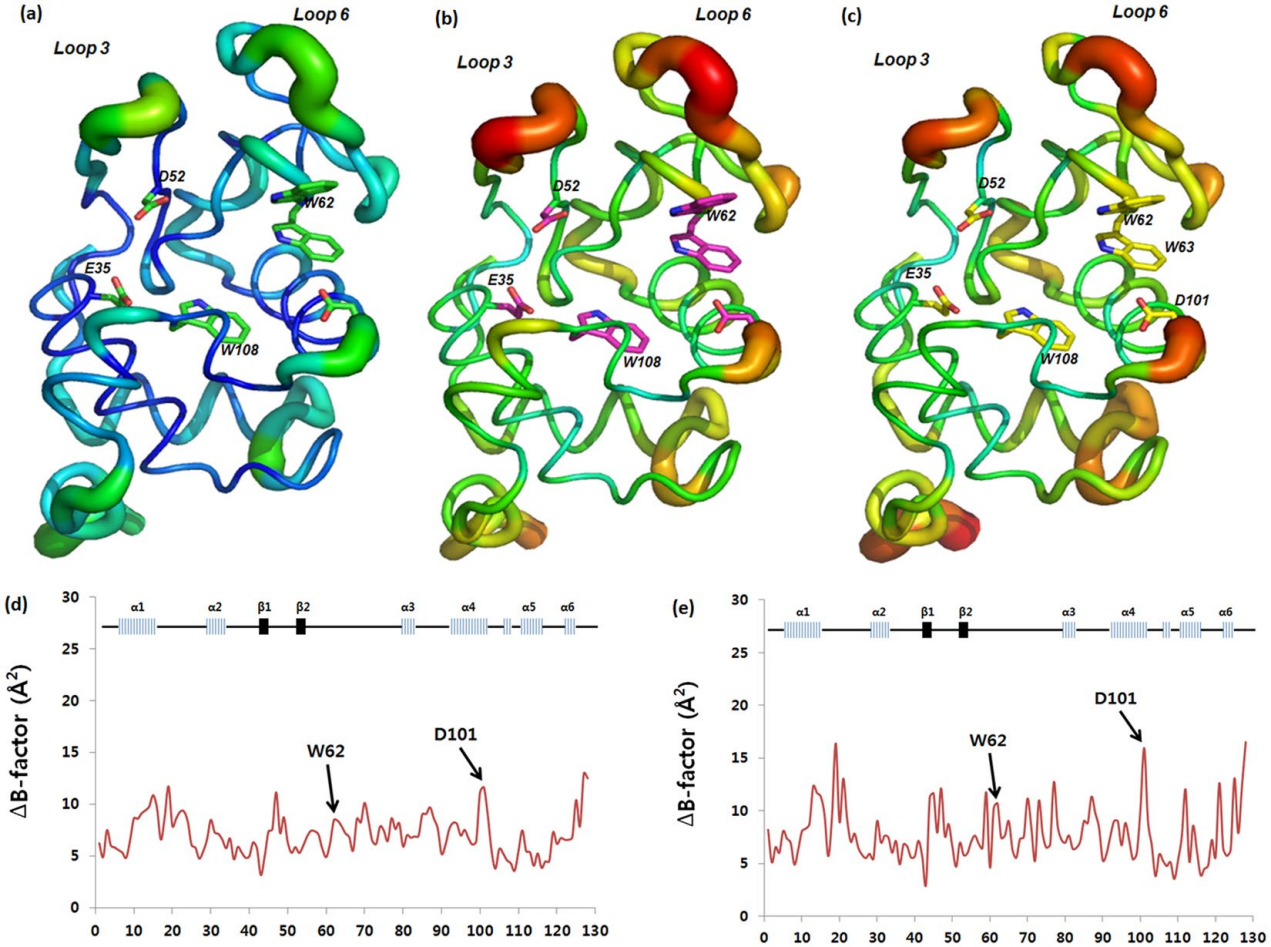
Thermodynamic change in enzyme structure after the 12 min plasma treatment analyzed by X-ray crystallography. (**a**) Control (without treatment); (**b**) Air-DBD; (**c**) N_2_-APPJ; Note that the wider and red indicates higher B-factors while narrower and blue means low B-factors. (**d**) Average B-factor differences in lysozyme residues after Air-DBD and (**e**) Average B-factor differences in lysozyme residues after N_2_-APPJ.

The structural conformational differences that occurred in lysozyme before and after plasma treatment are shown in Fig. 6a–c. We further evaluated the average B-factor to observe the thermodynamic changes of lysozyme after the plasma treatment. Figure 6d,e shows the differences of the B-factors between the control lysozyme (without plasma irradiation) and after 12 min of air DBD treatment. Among the catalytic residues, D101 and W62 showed larger B-factor differences than the average difference values (Fig. 6d). Similarly, differences for N_2_ feeding gas APPJ-treated lysozyme B-factor values and those of the control were calculated, and the results are shown in Fig. 6e. The patterns of the B-factor changes were similar between air and N_2_ treatment, but N_2_ irradiation showed overall larger differences compared to the air plasma treatment, indicating that the N_2_ plasma has a greater effect on the protein’s dynamics.

## Discussion

Overall, we observed that the lysozyme structure showed different changes when applying different types of plasma sources and feeding gases. The CD results showed that after treatment with DBD plasma, the proportion of the α-helix decreased and the proportion of the β-sheet increased in the lysozyme structure. By contrast, after the treatment with APPJ, the proportion of the α-helix increased and the proportion of the β-sheet decreased in the lysozyme structure, irrespective of the applied feeding gas. Comparison of the lysozyme structure changes after the DBD or APPJ plasma treatments using N_2_ and air as feeding gases showed that there were less structural changes when air was used as the feeding gas as compared to N_2_. These results supported the findings of our previous study, in which we observed more structural changes for N_2_ feeding gas plasma as compared to other feeding gases^26^. Furthermore, as we increased the time of treatment from 8 min to 12 min, the amount of structural changes observed was also increased; hence, treatment time plays an important role in inducing structural changes. If we take a close look at the reactive species generated by DBD and APPJ, we observed that the feeding gas plays an important role in RS generation as well as there was a difference of energy between two plasma sources. Another reason might be impact of the plasma plume on the enzyme structure. The plasma plume impact was higher for the APPJ as compared to DBD, as the APPJ plume directly hit a particular area, but for DBD there was uniform treatment on the wide area. These reasons may be responsible for the different types of structural changes for lysozyme after APPJ and DBD treatments.

We also conducted fluorescence spectroscopy to examine the modifications of the tertiary structure of lysozyme by detecting the Trp fluorescence pattern. We observed a quenching in the fluorescence intensity after the plasma treatment with no change in the wavelength. The quenching increased as we increased the time interval for both the DBD and APPJ plasma treatments with both feeding gases. More quenching was observed for the 12-min treatment as compared to the 8-min treatment for both plasma devices with both feeding gases. The quenching in fluorescence intensity was due to the modification of the Trp group or Trp surroundings. These results were also supported by a previous study of Takai *et al*., who treated lysozyme with He/O_2_ gas low-frequency plasma and observed fluorescence quenching. As suggested by a previous study^30^, the majority of the fluorescence emission was due to Trp62 and Trp108 in the native lysozyme structure, with a small contribution to the fluorescence spectra from residues other than Trp that were located near methionine sulphurs or cysteine^39^.

We further examined the crystal structure of lysozyme after Air-DBD and N_2_-APPJ treatments for 8 and 12 mins. We observed that loop 3 and loop 6 were mainly affected by the treatment with Air-DBD and N_2_-APPJ for 12 min treatments, while there was no structure change observed for 8 min treatment as compared to control, as shown in Fig. 6. Even the substrate binding site changed after the 12 min plasma treatment but no structural changes were observed for 8 min treatment. Evaluation of the enzyme activity also supported the results of the structural analysis, in which the N_2_ APPJ treatment showed the greatest effect on decreasing lysozyme activity. Moreover, the lysozyme activity was mainly affected by the APPJ treatment as compared to the DBD treatment for both feeding gases. This supports our crystal data that the substrate binding site was disturbed a lot after the plasma treatment and N_2_-APPJ caused higher disturbance to the substrate binding site. Finally, we checked the thermodynamic of lysozyme before and after the treatment using CD thermal analysis and calculation of the average B-factor. We observed the T_m_ was highly decreased following the N_2_-APPJ treatment and was least decreased following the Air-DBD plasma treatment in a treatment time-dependent manner. Further, the analysis of the average B-factor also showed that N_2_-APPJ induced the greatest change to the thermodynamics of lysozyme.

Overall, these experimental data demonstrated that APPJ has the greatest influence on both the lysozyme structure and activity, and that N_2_ is the most effective feeding gas for plasma treatment. To understand the mechanism of action of plasma, we considered the amount of energy and RS generated from the different plasma sources with the different feeding gases. Recently, Keider’s group^40^ reported that the change in plasma parameters affected cell viability due to variation in the ROS/RNS level of the medium. Similarly, in our previous work, we observed that different feeding gases resulted in different levels of structural changes, and among all the gases tested, N_2_ plasma showed the strongest effect^26^. One of the reasons for this result is that N_2_ plasma is excited by the N_2_ at a metastable level, N_2_(A_3_∑_u_^+^)^41^, which dissociates water molecules, thereby generating ^·^OH and ^·^H, as shown in equation (1). In addition, H_2_O molecules are dissociated by the excited N atom to generate two OH radicals:

1 $${{\rm{N}}}_{2}({{\rm{A}}}_{3}{\sum }_{{\rm{u}}}^{+})+{{\rm{H}}}_{2}{\rm{O}}\to {\rm{OH}}+{\rm{H}}+{{\rm{N}}}_{2}$$

2 $${{\rm{N}}}_{2}+{\rm{e}}\to 2{\rm{N}}+{\rm{e}}$$

3 $$2{\rm{N}}+2{{\rm{H}}}_{2}{\rm{O}}\to {{\rm{N}}}_{2}+2{\rm{OH}}+2{\rm{H}}$$

In air plasma, water molecular dissociation also occurs due to the reaction of N_2_(A_3_∑_u_^+^). However, in the air, OH molecules interact with O as shown in equation (4), and H reacts with O_2_ as shown in equation (5):

4 $${\rm{OH}}+{\rm{O}}\to {{\rm{O}}}_{2}+{\rm{H}}$$

5 $${\rm{H}}+{{\rm{O}}}_{2}\to {{\rm{HO}}}_{2}$$

OH is further eliminated by the reactions shown in equations (6) and (7):

6 $${\rm{OH}}+{\rm{OH}}\to {{\rm{H}}}_{2}{{\rm{O}}}_{2}$$

7 $${\rm{OH}}+{{\rm{HO}}}_{2}\to {{\rm{H}}}_{2}{\rm{O}}+{{\rm{O}}}_{2}$$

Therefore, the presence of oxygen atoms in air plasma as seen in the OES spectra (Figure S3) reduced the OH radical density, resulting in a lower concentration of H_2_O_2_ formed. Consequently, we observed that N_2_ plasma had the highest level of H_2_O_2_ and OH radicals generation followed by Air plasma (Figure S1). These results also support recent work reported by our group and another research group, in that N_2_ gas plasma produced more OH radicals than other gases^26, 42^. Therefore, this is likely the main reason for the observation of the reduced amount of structural changes in the Air plasma. These reductions in the OH and H_2_O_2_ generation for air plasma as compared to N_2_ plasma were found irrespective of the plasma sources, as shown in Figure S1. However, for air plasma some additional reactions could also occur such as

8 $${\rm{O}}+{\rm{N}}\to {\rm{NO}}$$

9 $${\rm{NO}}+{\rm{O}}\to {{\rm{NO}}}_{2}$$

10 $$2{{\rm{NO}}}_{2}+{{\rm{H}}}_{{\rm{2}}}{\rm{O}}\to {{\rm{NO}}}_{2}^{-}+{{\rm{NO}}}_{3}^{-}+2{{\rm{H}}}^{+}$$

11 $$4{\rm{NO}}+2{{\rm{H}}}_{2}{\rm{O}}+{{\rm{O}}}_{2}\to 4{{\rm{H}}}^{+}+4{{\rm{NO}}}_{2}^{-}$$

These reactions result in the formation of NO_2_^−^ and NO_3_^−^, which was evident by the observation of a slight decrease in pH, as shown in Figure S2. However, for the N_2_ plasma, there was a slight increase in the pH as compared to air plasma, likely due to the formation of NH_3_, as suggested in equation (12):

12 $${\rm{N}}+3{\rm{H}}\to {{\rm{NH}}}_{3}$$

These experimental results shows, that plasma can modify the structure along with change in the substrate binding site. The structural and catalytic modification for lysozyme is more for the N_2_-APPJ as compared with Air-APPJ.

## Conclusion

In this study, we present the first X-crystallographic data to, clearly illustrate the structural changes in lysozyme (loop 3 and loop 6) along with the change in the substrate binding site (W62, D101 and W108) after plasma treatment. Our CD, fluorescence, thermodynamic analysis and enzyme activity results clearly show that N_2_ feeding gas disturb the structure more than Air feeding gas (when the distance of treatment between plasma device and solution keep constant for treatments, additionally plasma characteristics such as gas flow rate, treatment time and voltage keep constant).

## Experiment Section

### Materials

Lysozyme and other chemicals were supplied by Sigma-Aldrich Chemical Co. (USA). All chemicals and reagents were used without further purification. H_2_O_2_ was measured using an titanyl ion^8^, and an NO^8^ was detected using 4-amino-5-methylamino-2′,7′-difluorofluorescein (DAF-FM) as described in our previous study. OH was also measured using terephthalic acid (20 mM) dissolved in saline exposed to plasma and the fluorescence of treated solutions was measured at an excitation wavelength of 310 mm and an emission wavelength of 425 mm^8^. The obtained fluorescence values were normalized with control values.

### Fluorescence spectroscopy

The fluorescence spectroscopy instrument used for measuring the fluorescence intensity in the present investigation is similar to that depicted in our previous report^29^. Steady-state fluorescence measurements were carried out with a PerkinElmer LS 55 fluorescence spectrometer. The excitation wavelength was fixed at 280 nm to determine the contribution of the degradation of the heme group from the overall fluorescence emission. The slit widths for excitation and emissions were set at 10 and 10 nm, respectively. The enzyme concentration for this experiment was 1 mg/ml.

### Circular dichroism spectroscopy

CD spectroscopic analyses^43, 44^ were performed using a J-815 spectrophotometer (Jasco, Japan) equipped with a Peltier system for controlling the temperature. (1S)-(+)-10-Camphorsulfonic acid (Aldrich, Milwaukee, WI, USA) was utilised for CD calibrations, exhibiting a molar extinction coefficient of 34.5 M/cm at 285 nm, and 2.36 M/cm molar ellipticity (θ) at 295 nm. Samples were pre-equilibrated at the desired temperature for 15 min and the scan speed was fixed for adaptive sampling (error F 0.01) with a response time of 1 s and 1 nm bandwidth. The secondary structure of lysozyme was monitored by using 1.0 cm path length cuvette. The lysozyme concentrations for secondary structures determination was 0.1 mg/ml, and each spectrum was an average of six spectra. Each sample spectrum was obtained by subtracting the spectrum of the appropriate blank media without lysozyme from the experimental enzyme spectrum. The percentages of secondary structures were then calculated by using Jasco, Japansoftware. To measure the T_m_ values (the higher the transition midpoint (T_m_) when 50% of the biomolecules are unfolded, the more stable the molecule) from the CD data, we used lysozyme at a 1 mg/ml concentration.

### pH and temperature measurement

After plasma exposure in buffer for different interval of time, the pH and temperature of the buffer was measured using a pH meter (Eutech Instruments, Singapore) and Infrared (IR) camera (Fluke Ti100 Series Thermal Imaging Cameras, UK). All measurements were carried out in triplicate.

### Atmospheric Pressure Plasma Jet (APPJ)

The soft plasma comprised mainly of, electrodes, dielectrics, and a high-voltage power supply^26^. A commercial transformer for neon light was operated at 60 Hz as the AC power supply. The air and N_2_ gas (99.99% pure) flow rate was 1.0 l pm, and a plasma jet plume was ejected into the open air througha1mmhole. The input voltage was approximately 75 V (V_rms_ is 0.4 kV and I_rms_ is 20 mA), and the frequency was 23 kHz for N_2_ plasma. For air APPJ the input voltage was 75 V (V_rms_ is 0.6 kV and I_rms_ is 32 mA), and the frequency is about the 24 kHz.

The DBD plasma device consists mainly of electrodes, dielectric layer (silicon dioxide [SiO_2_]) hydration prevention layers of aluminum oxide (Al_2_O_3_), and a magnesium oxide (MgO) layer^11^. The electrode is 5 µm thick and 200 µm wide with a 200 µm electrode gap and of a ≈1 µm thick MgO layer. The thickness of the SiO_2_ dielectric layer is about 30 µm and the diameter of the plasma discharge area is about 60 mm. To prevent hydration during plasma discharge, Al_2_O_3_ was added below the MgO layer. To generate plasma, Ar gas was injected into the device with a flow rate of l l pm. A commercial transformer for neon light is operated at 60 Hz as the AC power supply. The input voltage was 140 V (V_rms_ is 0.9 kV I_rms_ is 1.5 mA), and the frequency for N_2_ plasma was about 16 kHz. For air DBD the input voltage was about 140 V (V_rms_ is 1.3 kV and I_rms_ is 7 mA), and the frequency was about the 16 kHz. TheOES spectra of the APPJ emission were recorded by the use of HR4000CG-UV-NIR (Ocean Optics, FL, USA) and an optical fiber (QP400-2-SR) with a diameter of 400 mm, in a humid free atmosphere (the vacuum chamber was fabricated to study the activity in a closed environment). UV spectra were detected over a wide wavelength range of 200–1100 nm without solution. The signal was accumulated for 3 min, and the data were analyzed using the Origin 8.0 software package. The 1 ml samples were treated at 3 mm distance from DBD plasma source edge and 6 mm distance from APPJ edge.

### Crystallization

Lysozyme purchased as lyophilized powder (Sigma-Aldrich Chemical Co.) was dissolved in 0.1 M NaOAc, pH 4.8 to make a 50 mg/ml lysozyme solution. Lysozyme solutions were treated with 8 min and 12 min of plasma irradiation, respectively, and each plasma-irradiated lysozyme solution was subjected to a crystallization trial along with a control lysozyme solution without plasma irradiation. Crystals were obtained in one day by the sitting drop vapor diffusion method by mixing 2 μl of protein solution and an equal volume of crystallization solution [30% (w/v) polyethylene glycol 5000, 1 M NaCl, 50 mM Na-acetate, pH 4.5].

### Data collection and structure determination

After soaking the crystals in a cryoprotectant solution (crystallization solution with 20% ethylene glycol as a cryoprotectant), X-ray diffraction data were collected at the beamline 17 A at the Photon Factory, Japan. The phase problem was solved by molecular replacement using the crystal structure of hen egg-white lysozyme (PDB code: 1DPX) as a search model, and iterative model building and structure refinement was performed using the Coot program and Refmac5 in the CCP4 program suite, respectively^45, 46^.

### Enzyme activity

The lysozyme activity assay was performed with *Micrococcus lysodeikticus* bacterial cells as a substrate for the lysozyme. Enzyme activity was monitored by following the decrease in optical density occurring during the reaction. Cells were dissolved in buffer [50 mM potassium phosphate, 0.02% sodium azide, pH 6.4]. Optical densities were measured on an Optizen 2120 UV spectrophotometer against a blank of potassium phosphate buffer.

### Statistical analysis

All values are represented as the mean ± S.D of the indicated five times of replicates. The significance values were calculated as compared to control. Statistical analyses of the data were performed using the Student’s t-test to establish significance between data points. Significant differences were judged at *P < 0.05 and **P < 0.01.

## Electronic supplementary material

Supporting Information
