# Supplementary material for: Structural and functional analysis of lysozyme after treatment with dielectric barrier discharge plasma and atmospheric pressure plasma jet
Source: Sci Rep. 2017 Apr 21;7:1027. doi: 10.1038/s41598-017-01030-w (PMC5430822; doi:10.1038/s41598-017-01030-w)
Supplement: Supplementary file 1 — Supporting Information [file 41598_2017_1030_MOESM1_ESM.doc]

**Supporting Information**

**Structural and functional analysis of lysozyme after treatment with dielectric barrier discharge plasma and atmospheric pressure plasma jet** Sooho Choi^1§^, Pankaj Attri^2§^, Inhwan Lee^1§^, Jeongmin Oh^1^, Ji-Hye Yun^1^, Ji Hoon Park^2^, Eun Ha Choi^2^, and Weontae Lee^1*^

*^1^Department of Biochemistry, College of Life Science & Biotechnology, Yonsei University,*

*Seoul, Korea 120-749.*

*^2^Plasma Bioscience Research Center / Department of Electrical and Biological Physics, Kwangwoon University, Seoul, Korea 139-701.*

**Figure S1:** Radicals generated with treatment of DBD and APPJ for Air and N_2_ feeding gases for 12 min. All values are represented as the mean ± S.D of the indicated five times of replicates. (a) OH radicals (b) NO radicals and (c) H_2_O_2_. (*denotes P < 0.05 and **denotes P < 0.01).

**Figure S2:** (a) pH and (b) Temperature change during the treatment with DBD and APPJ for Air and N_2_ feeding gases for 12 min.

**Figure S3:** Optical emission spectra of the Air DBD and Air APPJ

**Figure S4:** Enzyme activity of lysozyme after the treatment for 12 min with DBD and APPJ for Air and N_2_ feeding gases


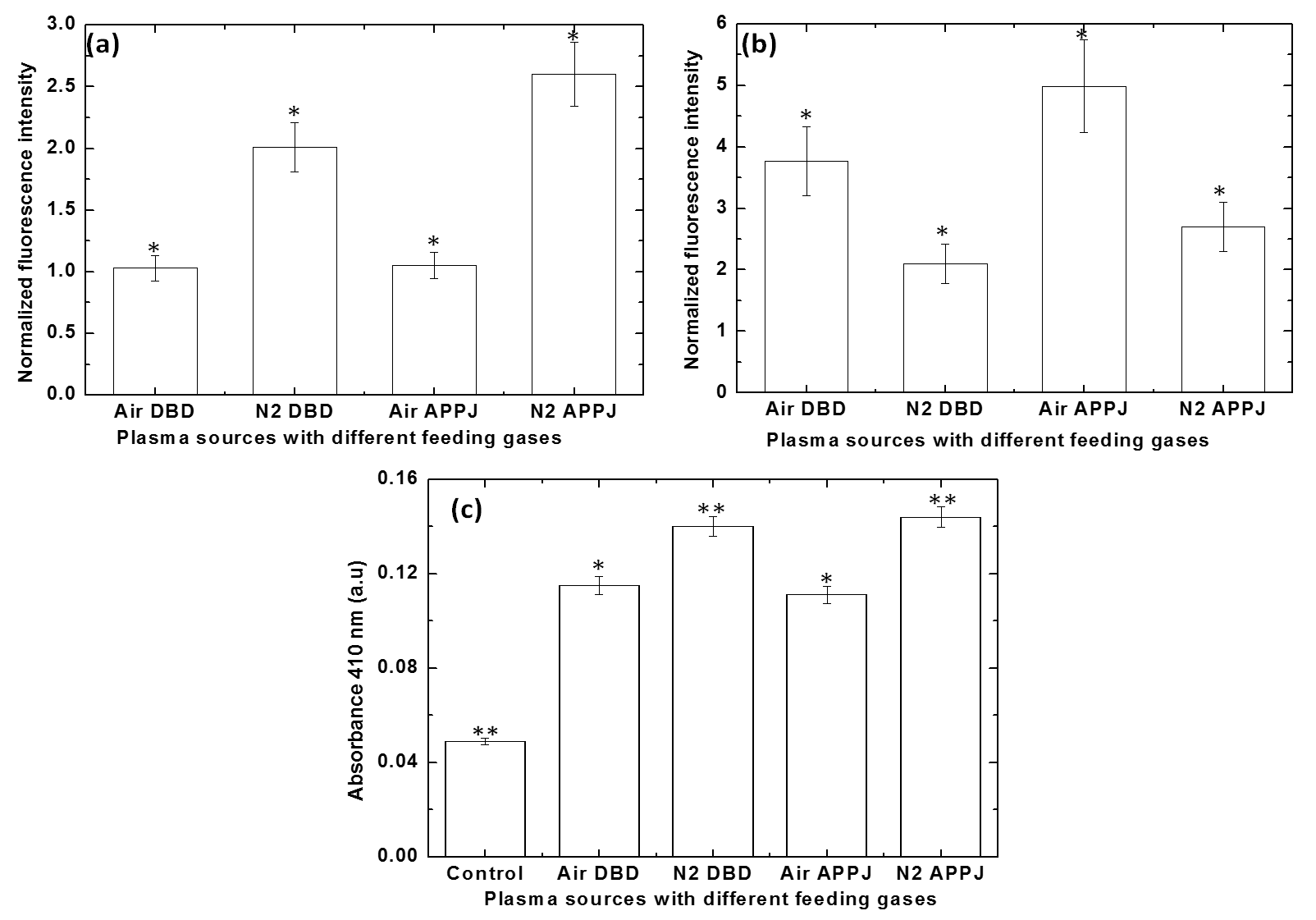
**Figure S1**

**Figure S2**

**
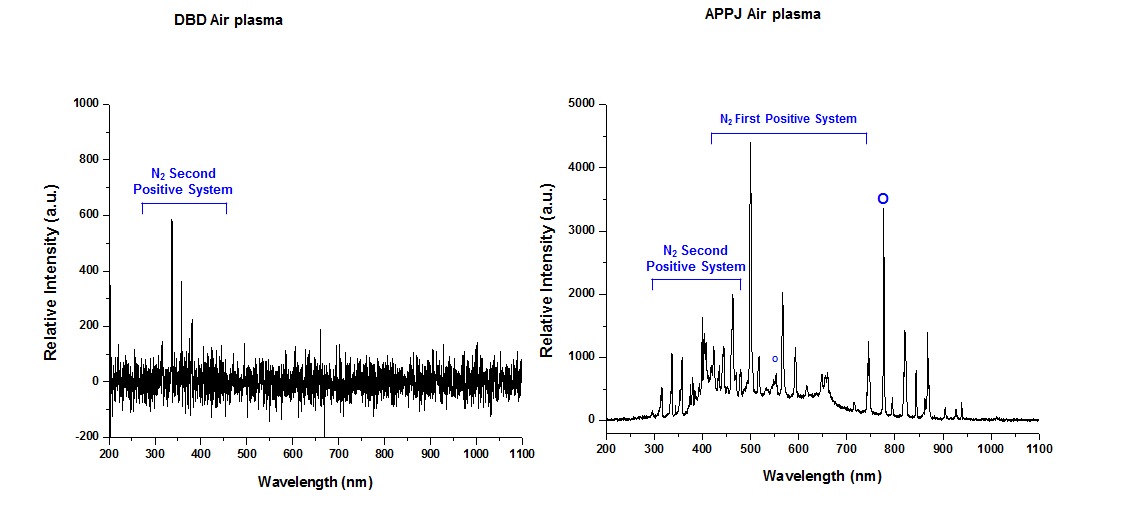
**

**Figure S3**

**
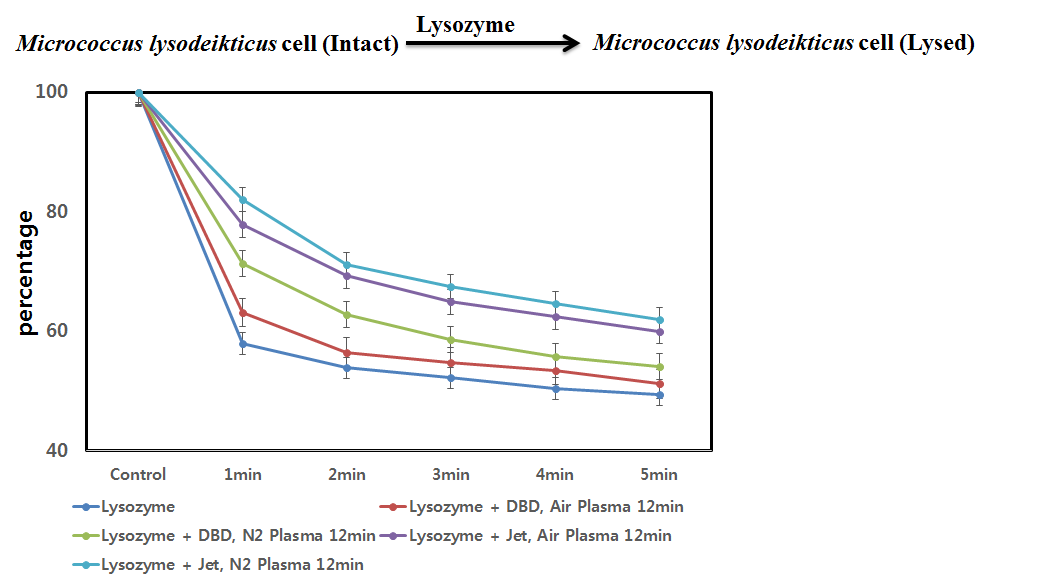
**

**Figure S4**

**Table S1. Secondary structure composition of Lysozyme, determined from Far UV CD spectra in different plasma sources with different feeding gases at 20 ^0^C.**

|  | α-Helix (%) | β-sheet (%) | Turn (%) | Random coil (%) |
| --- | --- | --- | --- | --- |
| Lysozyme | 51.3 | 16.1 | 0.0 | 32.6 |
| Lysozyme + Air DBD for 8min | 49.7 | 16.8 | 0.0 | 33.5 |
| Lysozyme + Air DBD for 12min | 48.3 | 17.8 | 0.0 | 33.9 |
| Lysozyme + N_2_ DBD for 8min | 31.0 | 27.0 | 0.0 | 42.0 |
| Lysozyme + N_2_ DBD 12min | 27.1 | 29.3 | 0.0 | 43.7 |
| Lysozyme + Air APPJ for 8min | 58.2 | 9.9 | 0.0 | 31.9 |
| Lysozyme + Air APPJ for 12min | 62.0 | 7.0 | 0.0 | 31.0 |
| Lysozyme + N_2_ APPJ for 8min | 59.5 | 9.0 | 0.0 | 31.4 |
| Lysozyme + N_2_ APPJ 12min | 70.4 | 1.5 | 0.0 | 28.6 |

**Table S2. Thermodynamic changes in the Lysozyme before and after plasma treatment.**

| **Samples** | **Melting Temperature**  **(T_m_ ℃)** |
| --- | --- |
| Lysozyme | 78.12 ± 0.4 |
| Air DBD 8min | 77.30 ± 0.6 |
| Air DBD 12min | 76.82 ± 0.5 |
| N_2_ DBD 8min | 76.12 ± 0.9 |
| N_2_ DBD 12min | 74.91 ± 1.4 |
| Air APPJ 8min | 77.25 ± 0.6 |
| Air APPJ 12min | 75.85 ± 0.7 |
| N_2_ APPJ 8min | 76.09 ± 0.5 |
| N_2_ APPJ 12min | 74.01 ± 0.5 |
